# Evaluation of psychological support for victims of sexual violence in a conflict setting: results from Brazzaville, Congo

**DOI:** 10.1186/1752-4458-3-7

**Published:** 2009-04-01

**Authors:** Sarah Hustache, Marie-Rose Moro, Jacky Roptin, Renato Souza, Grégoire Magloire Gansou, Alain Mbemba, Thomas Roederer, Rebecca F Grais, Valérie Gaboulaud, Thierry Baubet

**Affiliations:** 1 Epicentre, 8 rue Saint Sabin, 75011, Paris, France; 2 Hôpital Avicenne, Assistance Publique Hôpitaux de Paris, Université de Paris 13, 125 rue de Stalingrad, 93009 Bobigny, France; 3 Hôpital Cochin, Maison des adolescents, Université de Paris 5, 97 Bd de Port Royal, 75679, Paris cedex 14, France; 4 Médecins Sans Frontières France, 8 rue Saint Sabin, Paris, France; 5 Médecin Sans Frontières Switzerland, 78 Rue de Lausanne, CP 116, 1211, Geneva 21, Switzerland; 6 Faculté des Sciences de la Santé, Abomey-Calavi University, Centre National Hospitalier et Universitaire Hubert Koutoukou Maga, 03 BP 1890, Cotonou, Benin; 7 Makélékélé Hospital, Brazzaville, Hôpital de base de Makélékélé, BP 3219, Bacongo, Republic of Congo

## Abstract

**Background:**

Little is known about the impact of psychological support in war and transcultural contexts and in particular, whether there are lasting benefits. Here, we present an evaluation of the late effect of post-rape psychological support provided to women in Brazzaville, Republic of Congo.

**Methods:**

Women who attended the Médecins Sans Frontières program for sexual violence in Brazzaville during the conflict were selected to evaluate the psychological consequences of rape and the late effect of post-rape psychological support. A total of 178 patients met the eligibility criteria: 1) Women aged more than 15 years; 2) raped by unknown person(s) wearing military clothes; 3) admitted to the program between the 1/1/2002 and the 30/4/2003; and 4) living in Brazzaville.

**Results:**

The initial diagnosis according to DSM criteria showed a predominance of anxious disorders (54.1%) and acute stress disorders (24.6%). One to two years after the initial psychological care, 64 women were evaluated using the Trauma Screening Questionnaire (TSQ), the Global Assessment of Functioning scale (GAF) and an assessment scale to address medico-psychological care in emergencies (EUMP). Two patients (3.1%) met the needed criteria for PTSD diagnosis from the TSQ. Among the 56 women evaluated using GAF both as pre and post-test, global functioning was significantly improved by initial post-rape support (50 women (89.3%) had extreme or medium impairment at first post-rape evaluation, and 16 (28.6%) after psychological care; p = 0.04). When interviewed one to two years later, the benefit was fully maintained (16 women (28.6%) presenting extreme or medium impairment).

**Conclusion:**

We found the benefits of post-rape psychological support to be present and lasting in this conflict situation. However, we were unable to evaluate all women for the long-term impact, underscoring the difficulty of leading evaluation studies in unstable contexts. Future research is needed to validate these findings in other settings.

## Background

Sexual violence is not simply a consequence or side effect of war and displacement, but rather can be used as a deliberate tool of war [1]. Aside from medical consequences, such as sexually transmitted diseases or unwanted pregnancies, sexual violence, and rape in particular, may lead to long-lasting trauma and suffering. Sometimes this takes the shape of mental health disorders whereas at other times it surfaces in less obvious ways such as shame, guilt, sleeping problems, difficulties in daily functioning and withdrawal [2]. Ensuring appropriate care for victims of sexual violence involves comprehensive medical, social and psychological care [3-5]. According to the World Health Organization, care for victims of sexual violence should be guided by the individual's wishes and needs and provided sensitively in a coordinated and timely fashion to avoid the need for multiple service visits [3]. Although this is the approach used in most developed countries, some research shows that the mental health needs of patients are often the least well met in low resource or conflict settings [6]. Moreover, culture shapes the way post-traumatic symptoms are experienced and communicated in such contexts [7,8]. Because of this complexity, few studies have focused on psychological and psychiatric effects – in the short, medium or long-term – particularly in war and trans-cultural contexts. Whereas mental health support seems to be an essential component of post-sexual violence care, little is known about long-term benefits, particularly in low resource conflict contexts. To our knowledge, there is no published literature concerning the impact of post-rape psychological support in such settings.

Here, we report on the long-term follow-up of patients that received psychological support as part of the integration of mental health care into sexual violence medical services in the Republic of Congo. We aim to describe the short term consequences of rape in this conflict and transcultural context, and the impact of psychological care, just following rape and in the longer term.

## Methods

### Study Setting

The Republic of Congo experienced periods of intense conflict and population displacement during the 1993–2002 civil war. Ensuing violence after a coup d'etat in October 1997 resulted in more than 10,000 deaths in Brazzaville alone. Nearly one third of the capital's population fled the town, mainly to the Pool Region, southwest of Brazzaville, and was left without access to food and medical care [9-11]. A massive population displacement occurred between May 1999 and February 2000 with the populations' return to Brazzaville [10,11].

**Figure 1:**
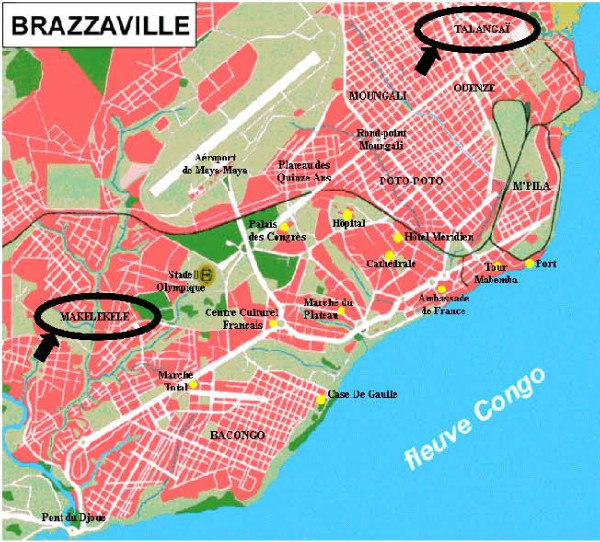
**Brazzaville map, Republic of Congo, 2004**.

The Non-Governmental Organization Médecins Sans Frontières (MSF) has been present in the Brazzaville area since 1998, providing medical care to the displaced population in the Pool region and the capital. As the national program had been interrupted because of the conflict, MSF started treating victims of sexual violence in Makelekele Hospital and Talangaï Hospital in Brazzaville, respectively in 2000 and 2003 (Figure 1). Patients in both programs were given free and anonymous medical care which included RU486 (the "morning after pill"), treatment for Sexually Transmitted Diseases (STD), HIV testing and prophylaxis as well as psychological support.

From March 2000 to June 2004, 1115 women were treated after sexual violence (925 in Makelekele hospital and 190 in Talangaï hospital). Among them, 86% (959) participated in post-rape psychological support. One year after the implementation of this programme, in August 2001, a preliminary assessment and the analysis of psychological symptoms during the initial care, based on the105 cases admitted at that time, showed a predominance of anxious disorders in 40% of the patients, with a prevalence of 14% for acute traumatic stress based on DSM-IV criteria [7].

### Participants

A total of 1115 persons were admitted to the MSF program between January 1, 2002 and April 30, 2003. Among them, 178 patients met the following criteria: 1) Women aged more than 15 years; 2) raped by an unknown person(s) wearing military clothes; 3) admitted to the MSF program between the 1/1/2002 and the 30/4/2003; and 4) and living in Brazzaville. Among these 178 women meeting the inclusion criteria, 19 refused psychological support at admission: we therefore have the initial diagnosis for 159 participants.

To locate study participants for follow up interviews, a trained social worker went to their home address. After obtaining oral informed consent, the social worker asked the victims to participate in a one hour interview with a psychologist to evaluate their current psychological state. If they agreed, an appointment was made for the interview. All psychological interviews took place between June 1, 2004 and July 15, 2004 at Makelekele Hospital, Brazzaville. They were all conducted by the same clinical psychologist with a Lingala or Lari translator. All women that were re-interviewed for evaluation were offered complementary assistance from one of the three psychologists working in the MSF programme in Makélékélé Hospital.

### Post-rape mental health care

The initial psychological intervention, conducted by a psychologist, made the initial diagnosis according to DSM-IV criteria [12]. Initial care included individual psychological support to support and improve the coping mechanisms of beneficiaries. Psychological care included:

a. provision of safe and empathetic environment so women could share their experiences;

b. active listening, allowing for expression of emotions, distress, fright, guilt, shame, anger, depressive and anxious affect;

c. allowing expression of personal views about events and distress, including cultural representations;

d. assessing familial and social consequences;

e. normalizing women's reactions to reassure that most of the women who have undergone such violence are experiencing similar reactions;

f. working on coping strategies; and

g. working on acceptance and development of future perspectives and plans.

The 178 included women underwent a median of 2 individual interventions [IQR 1–2], and 73% of them (n = 130) had between 1 and 4 interviews; 19 women refused to have any psychological support and accepted only medical care.

### Assessment Tools

Three different assessment tools were used to describe the psychological symptoms remaining one year after the trauma and their residual impact: The Trauma Screening Questionnaire (TSQ); an assessment scale to address medico-psychological care in emergencies (EUMP), designed for this study; and the Global Assessment of Functioning (GAF) scale.

The TSQ was administrated as a post-test questionnaire for long-term evaluation, taking approximately 15 minutes. The TSQ was developed and validated to identify reactions and likelihood of development of a Post Traumatic Stress Disorder (PTSD) in survivors of traumatic events [13]. Of the 10 questions asked during the TSQ, five are re-experiencing items and five are arousal items taken from the PTSD Symptom Scale-Self report version [14]. For each question, the answer is "yes" (being scored one) or "no" (being scored zero). The TSQ leads to a score between zero and ten; a PTSD is confirmed if the score is equal or more than six. The diagnosis of PTSD can be made only after more than one month of ongoing symptoms, and a later evaluation is useful because of delayed onset or a late exacerbation of the symptoms [15].

The GAF was administrated to all women attending the psychological support at initial care (pre-test), and re-administrated during the long-term evaluation (post-test). It reports the clinician's judgment of the woman's overall level of functioning and carrying out daily activities. This scale has been validated with psychiatric and non psychiatric patients in many settings, mainly in northern countries. [16-18]. It takes a few minutes to administer to the patient. The GAF scale considers psychological, social and occupational functioning on a continuum of mental health-illness, and is useful in measuring treatment impact. The clinician takes into account the number and intensity of signs and symptoms, and the consecutive alteration in global functioning. This scale is usually scored from zero to 100 (100 being the higher level of functioning in a large range of activities, without any symptoms). Due to the need for simplification, in our study, three levels of severity were defined: Low (scored from 61 to 100) corresponding to slight symptoms or functioning difficulties in the social, work or school sphere; Medium (scored from 31 to 60) corresponding to moderate or serious impairment in social, work or school functioning; and Severe (scored below 30) corresponding to a disability of functioning in all the spheres (for example someone remaining in bed, without any professional or social activity), with possible severe auto or hetero-aggressions.

The EUMP was used as a post-test for long-term evaluation (lasting approximately 30 minutes). It allows for the description of the early and delayed psychological consequences of trauma. It is a 33 items scale containing symptoms divided in 6 sections as follows: a) feelings during the event b) emotional reactions c) re-experiencing symptoms d) avoidance symptoms e) behavioural symptoms f) symptoms of difficulties in inter-personal relationships g) other associated symptoms (Cremniter D and Coq JM, unpublished). For each item, the score can be zero (no symptom), one (not intense and not frequent), two (quite intense and quite frequent) and three (very intense and very frequent). As this scale has not been yet internationally validated, unlike the GAF and the TSQ, results are presented through description of separate elements that could be revealed through this assessment.

Demographic, psychological and familial data about the patient at the date of the trauma were retrospectively collected from their medical form at initial care.

### Validity of instruments

All questionnaires were translated and back-translated from French to Lingala and Lari. To ensure acceptable cross-cultural adaptation, questionnaires were field-tested and semantic mistakes were discussed and corrected. The final version was validated by two senior psychiatrists. Internal consistency was measured by Cronbach's alpha for EUMP and TSQ, but not for GAF [19,20]. EUMP appeared to be highly internally correlated, with Cronbach's alpha = 0.89, whereas TSQ was less internally consistent (Cronbach's alpha = 0.68) [21]. Assuming that PTSD can indeed be considered as a more severe clinical manifestation than adjustment disorder, GAF seems to be measuring appropriately mental distress and functionality (Kruskal-Wallis p = 0.0005).

### Data analysis

All data was collected in Makelekele Hospital and entered into an EpiInfo 6.04d database (CDC Atlanta). Analysis was conducted using Stata 8.2 (Stata Corporation, College Station, Texas). Medians are given with inter-quartiles range [25%–75%]. Medians were compared using the Kruskal-Wallis test. Percentages were compared using the chi2 test.

The analyzed variables concerned the characteristics of the patient at admission to the program and at the long-term evaluation, the initial psychological diagnosis, and the psychological state evaluation using the TSQ, the EUMP and the GAF score.

### Ethical considerations

The Comité de Protection des Personnes of Université de Paris Nord granted an exemption for the investigators to conduct the data analysis with the previously collected data. Study protocol and objectives were explained to participants in French or in the local language prior to their inclusion and oral informed consent was obtained.

## Results

### Patient characteristics

**Figure 2:**
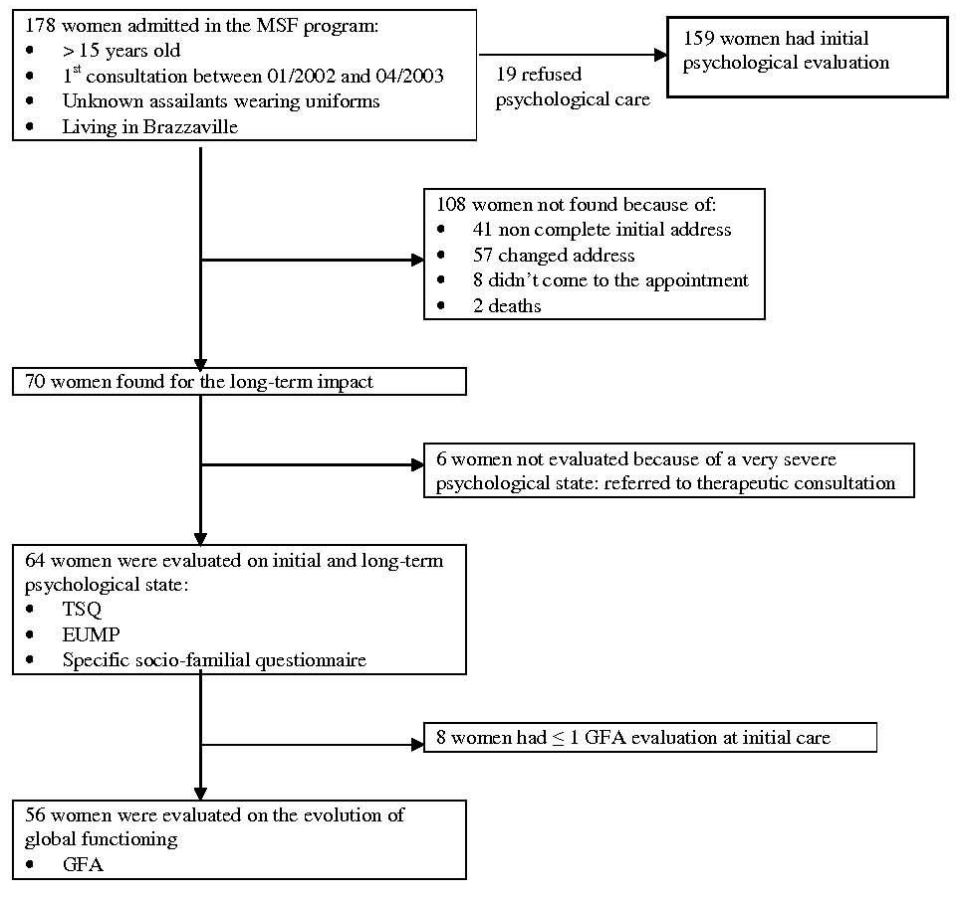
**Flow chart of included women, June 2004, Brazzaville, Republic of Congo**.

A total of 178 women admitted in the MSF sexual violence program met the inclusion criteria. Among them, 108 could not be evaluated one to two years later because they were not found: 41 (23%) had an incomplete or unknown address on their initial medical form, 57 (32%) changed address and were not found, eight (4.5%) were visited by the social worker at home but did not come for their appointment at the Makelekele Hospital, and two HIV-infected patients had died. Therefore, 70 out of the 178 women initially included (39.5%) were evaluated by a psychologist for the long-term psychological assessment (Figure 2).

Among these 70 women who were found to undergo the long-term impact interview with the psychologist, six (8.6%) could not be evaluated using the TSQ and the EUMP because of a severe psychological state. Four of these six women had been diagnosed as having an acute stress disorder during the initial psychological care and the two others presented initially anxious disorders.

There was no difference between the 70 women found for evaluation in the study and the 108 lost to follow-up in terms of age, number of attackers, delayed admission in the program after rape, or presence of traumatic lesions (p > 0.52). Differences between these two groups concerned the number of initial psychological consultations. The 70 evaluated women underwent a median of three interviews [IQR 2–4], whereas the 108 not evaluated in this study underwent a median of two interviews [IQR 1–3] (p = 0.001). The frequencies of reported pregnancies due to rape were also different (18.6% in evaluated women compared with 5.6% in those lost to follow up, p = 0.006) (Table 1).

**Table 1:** Characteristics of long-term evaluated and non-evaluated women meeting the inclusion criteria, June 2004, Brazzaville, Republic of Congo

	**Women evaluated**	**Women not evaluated**	**Total**	
	**n = 70**	**n = 108**	**n = 178**	
Age (years)	27 [18 – 36]	25 [19 – 36]	26 [19 – 36]	p = 0.86*
Number of attackers	2 [1 – 3]	2.5 [1.5 – 4]	2 [1 – 4]	p = 0.63*
Time between sexual violence and admission in the programme (weeks)	6 [2 – 17]	4 [2 – 12]	4 [2 – 13]	p = 0.52*
Number of psychological interviews	3 [2 – 4]	2 [1 – 3]	2 [1 – 4]	p = 0.001*
Pregnancy	13 (18.6%)	6 (5.6%)	19 (10.7%)	p = 0.006**
Presence of traumatic lesion after assault	16 (22.9%)	23 (21.3%)	39 (21.9%)	p = 0.81**

* Using Kruskal-Wallis non parametric test to compare medians

** Using chi2 test to compare percentages

Out of the 178 selected women, 121 were single (with or without children) when raped. Concerning the 70 women that were found for late evaluation in the study, 68.6% (48/70) and 75.7% (53/70) were single respectively at admission in the post-rape programme and when long term evaluated.

### Initial diagnosis

Among the 178 women, 19 did not have psychological support just after the sexual violence: they only accepted the medical care and refused to undergo any interview with the psychologist; they therefore had no psychological initial evaluation. The diagnosis according to DSM-IV criteria for the remaining 159 is described in Table 2. There was no significant difference in initial diagnosis between the 70 women that participated in the long-term evaluation and the 108 lost to follow-up (p = 0.9).

**Table 2:** Initial diagnosis (DSM-IV) for the 159 women interviewed by a psychologist when admitted in the program (evaluated or non evaluated for long-term impact) 2002/2003, Brazzaville, Republic of Congo

**Initial diagnosis**	**Evaluated for long-term impact**		**Non evaluated for long-term impact**		**Total**	
	**n**	**%**	**n**	**(%)**	**n**	**(%)**
Adjustment disorder	2	3	3	3.2	5	3.1
Acute stress disorder	14	21.2	25	26.9	39	24.6
PTSD	2	3	3	3.2	5	3.1
Severe depressive episode	6	9.1	8	8.6	14	8.8
Anxious disorder (generalized anxiety, panic disorder, phobic disorder...)	39	59.1	47	50.6	86	54.1
Other or not precised	3	4.6	7	7.5	10	6.3

Total	66**	100	93	100	159	100

P = 0.9

* Among the 178 included women, 19 refused the initial psychological evaluation when admitted

* * Out of the 70 evaluated women for long-term impact, 4 did not have initial diagnosis (refused psychological support)

### Long-term impact evaluation

Social and familial repercussions were assessed by a specific questionnaire. A total of 68.6% of the patients lived alone at the moment of the violence (with or without child), whereas they represented 75.7% at the long-term evaluation (p = 0.16). One to two years after violence, 31.3% of the victims reported a quite intense detachment in familial, professional and social spheres. Notably, 34.3% of the patients did not tell their family about their aggression.

During the interview conducted by the psychologist, the TSQ was administrated to 64 women (Table 3). Arousal symptoms were the most frequently reported, in particular 43.8% of the women reported a "heightened awareness of potential dangers to themselves", 37.5% reported "irritability or outbursts of anger" and 26.6% presented "difficulty falling asleep or staying asleep". The most reported re-experiencing items were "upsetting thoughts or memories about the event that have come into their mind against their will" (40.6%), and "feeling upset by reminders of the event" (26.6%).

**Table 3:** Reactions observed amongst the 64 women evaluated with the Trauma Screening Questionnaire, June 2004, Brazzaville, Republic of Congo

	Presence of reaction	
	n	%
**Re-experiencing symptoms**		
Upsetting thoughts or memories about the event that have come into your mind against your will	26	40.6
Upsetting dreams about the event	5	7.8
Acting or feeling as though the event were happening again	1	1.6
Feeling upset by reminders of the event	17	26.6
Bodily reaction when reminded of the event	5	7.8
**Arousal symptoms**		
Difficulty falling or staying asleep	17	26.6
Irritability or outbursts of anger	24	37.5
Difficulty concentrating	10	15.9
Heightened awareness of potential dangers to yourself or others	28	43.8
Being jumpy or being startled at something unexpected	0	0.0

The TSQ explores existence of PSTD: only two patients out of the 64 that could be evaluated (3.1%) met the needed criteria (score equal to or above 6) to make this diagnosis. The association of arousal symptoms was the most frequent with 15.6% of the woman combining at least four symptoms in this category.

The EUMP evaluation findings are consistent with the TSQ results concerning re-experiencing symptoms, present in 40.6% of the women. It brought out the existence avoidance (phobic manifestations and avoidance of the place where the assault took place and of men wearing military clothes) and somatic complaints, reported as quite intense by respectively 32.8 and 18.7% of the women. The most frequent symptom associated during evaluation with the EUMP was sexual disorders, reported as been quite or very intense by 32.9% of the women (Table 4).

**Table 4:** Symptoms reported by the 64 women evaluated with the EUMP, June 2004, Brazzaville, Republic of Congo

**Associated symptoms**	**No symptom**		**Not intense**		**Quite intense**		**Very intense**		**Unknown**	
	n	%	n	%	n	%	n	%	n	%
Changes in eating behaviour	56	87.5	5	7.8	1	1.6	0	0	2	3.1
Fatigue	37	57.8	21	32.8	5	7.8	0	0	1	1.6
Anhedonia	48	75	9	14.1	6	9.4	0	0	1	1.6
Somatic complaints	35	54.7	17	26.6	12	18.7	0	0	0	0
Sexual dysfunction	28	43.8	5	7.8	17	26.6	4	6.3	10	15.6
Sleep problems	34	53.1	23	35.9	7	10.9	0	0	0	0
Hypervigilance	49	76.6	10	15.6	1	1.6	0	0	4	6.3
Avoidance symptoms: Phobia/Obsession	13	20.3	27	42.2	21	32.8	1	1.6	2	3.1

The evolution of the patient's psychological state between the initial care and the long-term evaluation was assessed by GAF. This evaluation was made for 56 of the 70 women interviewed who had undergone at least two psychological consultations with GAF evaluation during the initial care (comparison when only one early post-traumatic GAF evaluation did not appear to be reliable). The GAF evaluation at the first psychological consultation after the assault showed an extreme or medium severity in global functioning for 89.3% of the women. After at least two psychological consultations, the severity is mild for more than 71.4% of the patients. This decrease in frequencies of extreme/medium to low impairment of global functioning is significant (p = 0.04). When interviewed one to two years after the initial care, the benefit of the psychological support after sexual violence is fully maintained, since exactly the same number of women (40 = 71.4%) reports low severity in global functioning impairment (Table 5).

**Table 5:** Global functioning evaluation using the GFA, during the first and last interview at initial care, and 1 to 2 years after sexual violence management, in the 56 women who benefit at least from 2 initial psychological consultations, June 2004, Brazzaville, Republic of Congo

**Global functioning impairment**	**GFA evaluation during the first interview**		**GFA evaluation during the last interview**		**GFA evaluation after 1 to 2 years**	
**n = 56**	n	%	n	%	n	%
Low impairment	6	10.7	40	71.4	40	71.4
Medium impairment	28	50.0	13	23.2	14	25.0
Severe impairment	22	39.3	3	5.4	2	3.6

## Discussion

Our findings suggest that benefits of post-rape psychological support, integrated in a global care, are present and lasting in this conflict context. In more than 2/3 of the patients, the GAF showed a clear improvement of psychological state between the first and the last psychological support during the initial management. These benefits were apparent with few interviews (median 3), and persisted afterward, supporting the fact that short psychological intervention therapies, conceivable in such settings, have an impact on the long-term resilience. The TSQ was not used at admission as a pre-test questionnaire, so the impact of psychological care in terms of PTSD cannot be adequately assessed by its domains evolution. We can however notice that 24.2% of the women presented an acute stress disorder or a PTSD when admitted in the post-rape programme, whereas it is much lower one to two years after rape (3.1% of the women who undergone the TSQ met a PTSD diagnosis criteria). Nevertheless, this improvement cannot be certainly attributed to the psychological care, as the spontaneous evolution could not be assessed by a control group.

Psychological reactions after sexual violence vary greatly, but overall people who experienced rape are more likely to develop PTSD than victims of any other crime [22,23]. However, TSQ results showed a low prevalence of PTSD of approximately 3%, confirming the low findings of a first evaluation of this program [7]. Considering that six women could not complete the TSQ because of a too severe psychological state, PTSD prevalence could have been underestimated. Nevertheless, even if we consider the extreme situation, that is all of them presenting a PTSD, the prevalence would be 11.4% (8/70). These results are very different from European or North American studies where post-rape PTSD prevalence was much higher, between 60 and 80% [24-27]. Some authors underline that PTSD, as defined in DSM-IV, cannot constitute or gather all the consequences of psychological trauma [27]. Moreover, DSM-IV has been developed for western-occidental psychiatry, and may not have the same validity in conflict contexts in Africa. There has been a tendency in Western psychiatric research to focus exclusively on PTSD when describing the psychological consequences of violence. Understanding human responses to extreme experiences solely in terms of PTSD has serious shortcomings. Nevertheless, PTSD prevalence in traumatized population was found very high in conflict contexts in Africa. Different studies conducted with war-affected Ugandans showed 40% to 54% of PTSD prevalence [28,29]. Others conducted with Internally Displaced People (IDP) in Kenya or with Sudanese refugees found respectively 80.2% and 40.1% of PTSD prevalence in the highly traumatized population [30,31]. There is ample evidence in support of the fact that Western conceptualizations of PTSD have validity in Africans, and that Africans can and do show symptoms of PTSD [32-34]. The low PTSD prevalence in our study is therefore interesting. African populations living in war contexts seem to develop PTSD as defined by DSM. This suggests that the low prevalence of PTSD in our study could be partly due to the initial psychological support. This hypothesis requires further evaluation, given that the aforementioned studies on PTSD in African population did not focus on victims of rape. At the contrary, the frequency of sexual dysfunction (1/3) is consistent with the results of European or American studies that report such disorders in 25% to 60% of sexual violence victims [27,35].

Several limitations require comment. Out of the 178 women meeting the inclusion criteria, 108 were lost to follow up, because they had an initial incomplete address or they had moved. Their characteristics when entering the program were similar to those participating in the study in terms of age, number of assailants, delay between rape and admission in the program and post-rape physical injuries. Nevertheless, it is not possible to extrapolate our findings to all the women that were admitted in the program. Notably, among the 70 women that could be evaluated, the proportion of unwanted pregnancy due to rape was higher than for those lost to follow up. This may be due to an increased need of socio-medical and psychological support in post-rape pregnant women. It is troublesome that more than half of the selected women could not be found because of the context (conflict and population displacement that did not allow to later locate the patients), and this leads to concerns regarding the generalizability of these findings. Nevertheless, this study, based on the interviews of 70 women admitted in a program which was not designed for long-term follow-up, provides the first long-term evaluation in this context.

The implementation of this study raised questions about the cultural appropriateness of the diagnosis process. The likelihood of cultural response bias to the questionnaires cannot be excluded. Moreover, there was no evidence of the cultural validity of our scales in this context. To reduce this cross-cultural bias, we tried to improve the semantic validity of the questionnaires using a mixed French and Congolese team for translation and back-translation, which was validated by two senior psychiatrists, and adjusted after being field-tested. Internal consistency of our scales, using Cronbach's alpha, appeared to be acceptable for the TSQ, and high for the EUMP which revealed information about avoidance symptoms, psycho-somatic and sexual disorders prevalence that are not developed within TSQ or GAF. The GAF as the sole pre and post test is problematic as there is a subjective component, and its nature of looking at global functioning does not well characterize some of the long term impacts of violence exposures that we attempted to address with the other tests. GAF internal consistency could not be measured with our data. However, we found a correlation between GAF score, which was the criterion we used to assess long-term impact, and the different initial diagnosis. This suggests that it is an appropriate instrument to measure mental distress and functionality in this context.

Our study is based on the assumption, that DSM-IV disorders have diagnostic validity across cultures. PTSD and other trauma stress disorders seem to be a cross-cultural way of post-traumatic suffering; nevertheless we cannot dismiss the eventuality of cultural different responses to trauma, particularly late complications that were not assessed in our study. Moreover, this type of impact study cannot lead to causal links as conducting a randomized placebo controlled trial would not be conceivable or ethical. The spontaneous evolution of psychological state improvement without any psychological intervention could not therefore be assessed. Evidence concerning the evolution of PTSD over time in similar contexts is mixed. Some evidence from Mozambique in a conflict context suggests that PTSD rates go down spontaneously over time; other studies suggest otherwise [36-38]. These studies evaluated war traumatized population, and did not focus on woman victims of sexual violence. Finally, it is difficult to distinguish the specific impact of psychological support as the women in a large majority benefited of integrated medico-socio-psychological care (only four refused psychological support when entering the program, which does not allow comparative analysis).

## Conclusion

This study suggests that there is a long-term positive impact of psychological support among victims of sexual violence during conflict, and underscores the importance of a multidisciplinary care. This is a first step towards evaluating such interventions and highlights the need for further validation. There is a clear need to adapt diagnostic and assessment tools for psychological and psychiatric care in difficult contexts to ensure the appropriate care of victims of violence.

## Competing interests

The authors declare that they have no competing interests.

## Authors' contributions

SH had full access to all of the data in the study and takes responsibility for the integrity of the data and the accuracy of the data analysis. TB, JR, MRM and VG participated in the study concept and design. SH, VG, RS, TR and RFG performed the statistical analysis and interpretation of the data. SH, RS, JR, RFG, TB, MRM, AM and GG participated in the critical revision of the manuscript. All authors read and approved the final manuscript.
